# Effectiveness of medical supportive team for outpatients treated with sorafenib: a retrospective study

**DOI:** 10.1186/s40780-014-0005-0

**Published:** 2015-02-18

**Authors:** Makoto Kajizono, Megumu Aoyagi, Yoshihisa Kitamura, Toshiaki Sendo

**Affiliations:** Department of Pharmacy, Okayama University Hospital, 2-5-1 Shikata-cho, Kita-ku, Okayama 700-8558 Japan

**Keywords:** Sorafenib, Hepatocellular carcinoma, Renal cell carcinoma, Adverse drug reactions, Hand-foot skin reactions, Medical supportive team, Outpatient

## Abstract

**Background:**

It is well known that molecular-targeted drugs, of which sorafenib (Nexavar®) is one, differ from previous anticancer drugs and cause various unusual adverse drug reactions. Treatment with sorafenib causes adverse drug reactions such as hand-foot skin reactions, hypertension, and diarrhea. Physicians spend a lot of time monitoring adverse drug reactions to sorafenib in outpatients. As such, at Okayama University Hospital, pharmacists and nurses have organized a medical supportive team to help physicians in this regard. However, the effectiveness of interventions for sorafenib-treated outpatients by this medical supportive team remains unclear. The purpose of this study was thus to clarify the effectiveness of interventions for sorafenib-treated outpatients by this medical supportive team.

**Methods:**

We retrospectively studied 70 outpatients treated with sorafenib between May 2009 and December 2012 at Okayama University Hospital. These outpatients were classified into two groups, an intervention group (31 outpatients) and a non-intervention group (39 outpatients). We compared the duration of sorafenib treatment between these groups.

**Results:**

The duration of treatment with sorafenib was significantly longer in the intervention group than in the non-intervention group. No outpatients in the intervention group discontinued sorafenib due to adverse drug reactions such as hand-foot skin reactions or diarrhea.

**Conclusion:**

The duration of sorafenib treatment was significantly longer in the intervention group than in the non-intervention group. Our findings suggest that interventions by the medical supportive team consisting of health care professionals were effective in preventing the discontinuation of sorafenib.

## Background

Sorafenib (Nexavar®) is one of the oral molecular-targeted drugs used as systemic treatment in patients with unresectable hepatocellular carcinoma and advanced renal cell carcinoma [1-3]. It inhibits the activity of serine/threonine kinases of RAF, protein kinases of MEK and ERK, and blocks vascular endothelial growth factor receptors (VEGFR), platelet-derived growth factor receptors (PDGFR), the cytokine receptor c-KIT, and the receptor tyrosine kinase Flt-3 to inhibit tumor angiogenesis and tumor cell growth [4-6].

**Figure 1:**
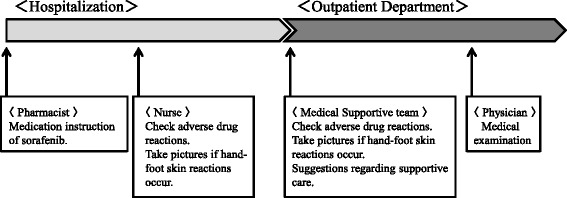
**Participation of the medical supportive team for those administered sorafenib in Okayama University Hospital.**

Molecular-targeted drugs were initially expected to cause less severe adverse drug reactions than previous anticancer drugs. However, they have been shown to cause various unusual adverse drug reactions. Sorafenib also causes many adverse drug reactions, such as hand-foot skin reactions, diarrhea, and hypertension [2,7,8]. Therefore, the dose of sorafenib has been decreased or sorafenib treatment has been discontinued [9,10]. In some hospitals, outpatients undergo interventions by a medical supportive team to prevent adverse drug reactions. We organized a medical supportive team consisting of pharmacists and nurse at Okayama University Hospital (Figure 1). Physicians do not have enough time in their limited consultations with outpatients to establish the adverse drug reactions of sorafenib. Pharmacists and nurse thus met with outpatients during the waiting time for blood sampling. We recorded suggestions on monitoring sheets regarding the problems encountered by patients, such as the grade of adverse drug reactions and afflictions. Furthermore, we also provided outpatients with some advice related to their problems, including adverse drug reactions or difficulties in daily life. If hand-foot skin reactions occurred, the team took pictures of the patient’s hand, foot, and skin, and posted them in an electronic medical record system. Thus, the medical supportive team supported physicians regarding the problems encountered by patients. Active participation of the medical supportive teams has been reported in the management of patients [11,12]. However, the effectiveness of interventions for patients by medical supportive teams has not been demonstrated. In our hospital, our medical supportive team initially intervented in cases with sorafenib treatment. In the present study, we examined whether a prolonged duration of sorafenib treatment in outpatients could be identified in association with interventions by the medical supportive team in our hospital.

## Methods

### Patients

We retrospectively screened outpatients with hepatocellular carcinoma and advanced renal cell carcinoma (70 patients) who had been treated with sorafenib between May 2009 and December 2012 at Okayama University Hospital. Outpatients were categorized according to age, gender, an Eastern Cooperative Oncology Group (ECOG) performance status of 0–2, previous therapy, metastatic site, and Child-Pugh classification for hepatocellular carcinoma patients. This study was approved by the Okayama University Graduate School of Medicine, Dentistry, and Pharmaceutical Sciences Ethics Committee (No. 650).

### The roles of the medical supportive team for sorafenib treatment

Our team is composed of two pharmacists (one Japanese Society of Pharmaceutical Health Care and Sciences-certified oncology pharmacist and one board certified pharmacist in oncology pharmacy) and one nurse (certified nurse in cancer chemotherapy nursing). One pharmacist and the nurse always intervene per patient, and the frequency of intervention is once every month. We check the medical situation, dose, and adverse events before a doctor’s examination. The main work of our team is to consider the advisability of administrating particular anti-cancer agents and other prescription drugs based on patient consultation, and to make recommendations to the physicians in charge.

### Outcome evaluation

We categorized outpatients into two groups based on whether or not they had been undergone on intervention by the medical supportive team in terms of the duration of sorafenib treatment. The intervention by the medical supportive team was decided according to the physician’s requirements. Patients were excluded from this study based on the guidelines for the appropriate use of sorafenib as follws: those with progressive disease, grade 2 hand-foot skin reactions more than 4 times, or grade 3 hand-foot skin reactions more than 3 times. When other non-hematotoxicities occurred, the sorafenib treatment had to be interrupted for more than 30 days. In addition, sorafenib treatment was also discontinued when the primary care physician considered continuation of the treatment to be unnecessary or a reduction in the sorafenib dose by 2 levels or more to be necessary. Adverse drug reactions were assessed according to the National Cancer Institute Common Terminology Criteria for Adverse Events (NCI-CTCAE), version 4.0.

### Statistical analysis

Categorical variables were compared by Fisher’s exact test, and the means of the two groups were analyzed by independent *t*-test. The duration of treatment was analyzed using Kaplan-Meier survival curves with log-rank survival comparisons. Significance was set at P <0.05. Statistical analysis was performed with the Statistical Package for the Social Sciences software, version 21 (IBM SPSS, Inc. Chicago, IL, USA).

## Results

### Patient characteristics

In this study, 72 outpatients were screened. Two patients were excluded from the analysis; one had not undergone an intervention by the medical supportive team and the other was only administered sorafenib for one day because of adverse drug reactions. Therefore, 70 outpatients were classified into two groups: an intervention group and a non-intervention group, according to interventions by the medical supportive team. No significant differences were observed in the baseline characteristics between the two groups, except for diagnosis (Table 1).

**Table 1:** **Patient characteristics**

	**Intervention group n = 31 (%)**	**Non‐intervention group n = 39 (%)**	**P-value**
Age	70	67	0.578
Range	51-86	28-88	
Gender			1.000
Male	27(87.1)	35(89.7)	
Female	4(12.9)	4(10.3)	
ECOG PS			0.645
0	17(54.8)	17(43.6)	
1	12(38.7)	19(48.7)	
2	2(6.45)	3(7.7)	
Diagnosis			0.001*
HCC	31(100)	27(69.2)	
RCC	-	12(30.8)	
Child-Pugh classification			0.233
A	29(93.5)	22(81.5)	
B	2(6.5)	5(18.5)	
**Previous therapy**			
HCC			0.186
None	7(22.6)	2(7.4)	
TACE	20(64.5)	22(81.5)	
HAIC	10(32.3)	3(11.1)	
RFA	10(32.3)	13(48.1)	
TAI	1(3.2)	1(3.7)	
Surgery	8(25.8)	3(11.1)	
Molecular-targeted therapy	2(6.5)	-	
RCC			0.001*
None		3(25.0)	
RFA	-	1(8.3)	
Interferon, Interleukin	-	5(41.7)	
Surgery	-	7(58.3)	
Molecular-targeted therapy	-	4(33.3)	
**Metastatic site**			
HCC			0.672
Lung	9(29.0)	4(14.8)	
Lymph node	9(29.0)	3(11.1)	
Bone	3(9.7)	1(3.7)	
Other	4(12.9)	4(14.8)	
RCC			0.001*
Lung	-	5(41.7)	
Lymph node	-	4(33.3)	
Bone	-	4(33.3)	
Liver	-	3(25.0)	
Other	-	6(50.0)	

*P < 0.05.

*Abbreviations:* ECOG PS: Eastern Cooperative Oncology Group performance status; HCC: hepatocellular carcinoma; RCC: renal cell carcinoma; TACE: transarterial chemoembolization; HAIC: hepatic arterial infusion chemotherapy; RFA: radiofrequency ablation; TAI: transcatheter arterial infusion.

### Duration of the sorafenib treatment

**Figure 2:**
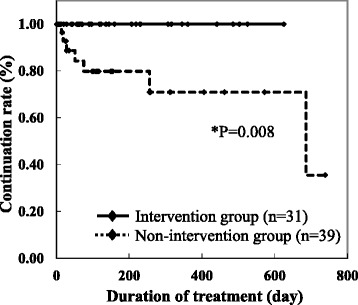
**Effect of interventions by the medical supportive team on the duration of sorafenib treatment in outpatients.**

The duration of the sorafenib treatment was significantly longer in the intervention group than in the non-intervention group (P = 0.008) (Figure 2). In the non-intervention group, 7 patients stopped using sorafenib due to adverse drug reactions that were potentially tolerable. Table 2 shows the reasons for the discontinuation of sorafenib in the non-intervention group. The dosage of sorafenib was not decreased from 800 mg/day in three cases. However, sorafenib treatment was interrupted in 3 outpatients to maintain a dose of 800 mg. Furthermore, one outpatient refused to continue the sorafenib treatment due to anorexia. The dose of sorafenib was decreased to 600 mg/day in a patient with hypothyroidism; however, inn this case, sorafenib treatment was discontinued without endocrine therapy such as thyroid gland auxiliary therapy.

**Table 2:** **Discontinuation of sorafenib in the non-intervention group (n = 7)**

**Adverse drug reactions (Grade)**	**Daily dose (mg)**	**Diagnosis**	**Previous therapy**
Hand-foot skin reaction (3)	800	hepatocellular carcinoma	TACE, RFA, Operation
Hand-foot skin reaction (3)	800	renal cell carcinoma	Interleukin
Hand-foot skin reaction (2)	800	renal cell carcinoma	Interferon, Sunitinib^a^
Hand-foot skin reaction (3)	200	hepatocellular carcinoma	TACE, RFA
Anorexia (2)	400	renal cell carcinoma	None
Hypothyroidism (2)	600	renal cell carcinoma	Surgery
Diarrhea (1)	800	hepatocellular carcinoma	TACE, RFA

^a^Molecular-targeted therapy.

*Abbreviations:* TACE: transarterial chemoembolization; RFA: radiofrequency ablation.

### Activities of the medical supportive team for outpatients

Mean daily doses of sorafenib were lower in the intervention group (486.5 ± 198.6 mg (median, 480.8 mg)) than in the non-intervention group (581.8 ± 196.0 mg (median, 600 mg)) (P = 0.048). Furthermore, no significant differences were observed in the mean initial doses of sorafenib between the intervention group (709.7 ± 170.0 mg (median, 800 mg)) and the non-intervention group (702.6 ± 170.9 mg (median, 800 mg)). The medical supportive team spent an average of 37.1 ± 11.1 min per outpatient (median, 40 min), and the total number of interventions was 146. A total of 61 interventions in the intervention group concerned suggestions on medication to physicians and outpatients. Table 3 shows the medical supportive team’s suggestions to physicians and patients. The incidences of adverse drug reactions, apart from those of grades 3–4, such as hand-foot skin reaction, diarrhea, anorexia, hair loss, thrombocytopenia, hoarseness, increased serum amylase, fatigue, and stomatitis, were higher in the intervention group than in the non-intervention group (Table 4).

**Table 3:** **Details of supportive care or suggestions by the medical supportive team**

**Category**	**For patients**	**For physicians**
Hand-foot skin reaction	11	3
Interaction of drugs	4	0
Diarrhea	3	8
Hypertension	2	6
Other adverse drug reactions	6	12
Introduction of social support for medical expenses	1	0
Discontinuation of sorafenib before surgery	0	1
Adherence	0	6

**Table 4:** **Incidence of adverse drug reactions**

**Adverse drug reactions**	**Intervention group (n = 31) n (%)**		**Non-intervention group (n = 39) n (%)**		**P-value (Any)**
Grade	Any	3 ~ 4	Any	3 ~ 4	
Hand-foot skin reaction^a^	23(74.2)	2(6.5)	19(48.7)	3(7.7)	0.049*
Diarrhea	21(67.7)	2(6.5)	5(12.8)	1(2.6)	< 0.001**
Hypertension	16(51.6)	6(19.4)	15(38.5)	5(12.5)	0.336
Anorexia	15(48.4)	1(3.2)	6(15.4)	-	0.002**
Hair loss	13(41.9)	-	5(12.8)	-	0.012*
Thrombocytopenia	11(35.5)	2(6.5)	4(10.3)	3(7.7)	0.018*
AST increased	11(35.5)	5(16.1)	6(15.4)	3(7.7)	0.091
Hoarseness	10(32.3)	-	4(10.3)	-	0.034*
ALT increased	9(29.0)	-	6(15.4)	3(7.7)	0.242
Serum amylase increased	9(29.0)	6(19.4)	3(7.7)	1(2.6)	0.026*
Fatigue	7(22.6)	-	1(2.6)	-	0.018*
Stomatitis	6(19.4)	-	1(2.6)	-	0.039*
Hypothyroidism	5(16.1)	-	3(7.7)	-	0.452
Blood bilirubin increased	5(16.1)	1(3.2)	3(7.7)	1(2.6)	0.452
Rash maculo-papular	4(12.9)	-	4(10.3)	-	1.000

^a^Palmar-plantar erythrodysesthesia syndrome (NCI-CTCAE).

*P < 0.05.

**P < 0.01.

## Discussion

In this study, the duration of treatment with sorafenib was significantly increased due to interventions by the medical supportive team for outpatients. The supportive team performed clinical management that included checking the adherence of patients to the use of moisturizing lotion for hand-foot skin reactions. If outpatients had not properly followed the instructions, we participated in pharmaceutical care including patient education for swabbing the ointment to avoid serious hand-foot skin reactions. Previous prospective or randomized trials had not determined the best management of hand-foot skin reactions caused by treatment with sorafenib. However, early use of a moisturizer was recommended to avoid hand-foot skin reaction symptoms [9,13,14]. Furthermore, the incidences of sorafenib-induced adverse drug reactions and hand-foot skin reactions were shown to be higher in Japanese patients than in European ones [10]. The medical supportive team performed clinical management that included four main activities; 1) checking adverse drug reactions before the physician’s consultation; 2) medication use evaluations based on laboratory data; 3) checking for adherence to sorafenib; and 4) providing drug information and lifestyle care points to patients in our hospital. It is hoped that the clinical activities of the medical supportive team will enhance the quality of sorafenib medication. In the present study, the discontinuation of sorafenib treatment by outpatients did not occur due to frequent communication with the medical supportive team. For this reason, the early stages of hand-foot skin reactions were detected by the medical supportive team.

On the other hand, information on the appropriate use of sorafenib suggests that treatment with it should be interrupted until recovery to Grades 0–1 when Grade 3 hand-foot skin reactions occur. Furthermore, if patients recover from this symptom, decreases of 400 mg in the dose (daily or alternate-day treatment) should be implemented when administration is resumed. The incidence of adverse drug reactions was higher in the intervention group than in the non-intervention group. These results together with the significantly low mean doses of sorafenib and higher incidence of adverse drug reactions in the intervention group suggest that the supportive team had a detailed interview with patients regarding adverse effects. Thus, interventions by the supportive team helped primary care physicians to reduce the sorafenib dose and appropriately prescribe supportive therapies. However, this study has the following limitations. Patients with advanced renal cell carcinoma were included in the non-intervention group. In patients with renal cell carcinoma, since molecular-targeted drugs other than sorafenib can be selected, there is a possibility that sorafenib treatment was readily discontinued. Some studies have suggested an association between prolongation of the sorafenib treatment period and clinical effects [15-17]. In addition, although there has been no evidence on the appropriate sorafenib dose in the Japanese, some studies have shown effects of sorafenib even at a small dose [18-20]. These findings suggest that measures against adverse effects taken by a supportive team prevent premature discontinuation of sorafenib treatment.

## Conclusions

In conclusion, the medical supportive team-implemented management of pharmaceutical care contributed to continuation of the use of sorafenib in outpatients. Furthermore, the activities of the medical supportive team are important not only for outpatients treated with sorafenib, but also for those with other molecular-targeted drugs used in other hospitals.

## Abbreviations

ECOG PS: Eastern cooperative oncology group performance status
HCC: Hepatocellular carcinoma
RCC: Renal cell carcinoma
TACE: Transarterial chemoembolization
HAIC: Hepatic arterial infusion chemotherapy
RFA: Radiofrequency ablation
TAI: Transcatheter arterial infusion
